# Editorial: Prescription and execution of therapeutic exercise programs in neurological disorders

**DOI:** 10.3389/fresc.2022.1060516

**Published:** 2022-11-21

**Authors:** J. Güeita-Rodríguez, J.B. Allendorfer

**Affiliations:** ^1^ Research Group of Humanities and Qualitative Research in Health Science, Department of Physical Therapy, Occupational Therapy, Rehabilitation and Physical Medicine, Rey Juan Carlos University, Madrid, Spain; ^2^ Departments of Neurology and Neurobiology, University of Alabama at Birmingham, Birmingham, AL, United States

**Keywords:** adherence, cognition, neurological disorders, social determinants of health, therapeutic exercise

**Editorial on the Research Topic** Prescription and execution of therapeutic exercise programs in neurological disorders By Güeita-Rodríguez J and Allendorfer JB. (2022) Front. Rehabilit. Sci. 3:1060516. doi:10.3389/fresc.2022.1060516

People with neurological disorders reduce their participation in daily activities due to their alterations, encouraging sedentary lifestyles. The levels of physical activity and participation at hospital and community levels after injury are very low (1), and there is a growing need for infrastructure and organizational systems of rehabilitation centers to increase their capacity and resources to combat the deleterious effects of sedentary behavior (2). Added strain on these systems from various psychosocial factors including depression and anxiety may also play an important role (3, 4).

In the last two decades, the prescription of therapeutic exercise programs in neurological disorders has been investigated with the aim of preventing damage to functions or structures, restoring function and participation in activities of daily living, preventing or reducing risk factors and optimizing health and sense of well-being. All this promotes participation in the activities that the person with neurological disorder performs in the real environment. Different benefits have also been described as secondary prevention, with neuroprotective effects in modifying the course of the disorder (5). Numerous studies show that exercise has beneficial effects on brain health in clinical populations (6, 7); however, adherence to exercise guidelines and recommendations is notoriously poor.

Part of the current scope of research in therapeutic exercise in neurological disorders is represented by five papers contributing to the Research Topic “Prescription and execution of therapeutic exercise programs in neurological disorders” from different areas.

## Exercise and adherence in aging patients

The physical activity recommendation by the World Health Organization for adults 65 and older is the same as for ages 18–64 years and for people with chronic conditions or living with disability, which is at least 150 min of moderate-intensity or at least 75 min of vigorous-intensity aerobic physical activity each week. For ages 65 years and older, there should also be an emphasis on functional balance and at least moderate-intensity strength training for three or more days each week (8). Overall, the studies published in this Research Topic shed new light on some important aspects of the field of exercise in aging, such as adherence in sedentary older adults and the benefits of physical activity and exercise in those at risk for cognitive decline.

A large body of scientific evidence supports that being physically active can prevent or delay the onset of cognitive decline and dementia. However, a critical obstacle is that, although many older adults recognize its health benefits, most do not exercise regularly. Clinical and scientific efforts are underway to establish specific exercise recommendations for cognitive brain health. But, an important knowledge gap is how to develop effective strategies to increase exercise participation and adherence in the aging population.

Cabral and colleagues developed a study to better understand the perspective of sedentary older adults regarding exercise. Participants revealed four main themes: (1) age and aging are determinants of exercise; (2) maintaining an active mind and autonomy are priorities, but planned exercise is not often considered as part of being “active”; (3) motivational challenges in exercise engagement; (4) the need for tailored recommendations. The authors presented a multidimensional model of exercise adherence to maximize brain health in older adults and suggested a set of tools and key questions to effectively assess sedentary older adults and translate current guidelines to the needs of the individual through the use of behavior change strategies (Cabral et al.).

Hinchman and colleagues examined the cognitive benefits in sedentary older adults at risk for cognitive decline of a moderate-high intensity aerobic exercise program (in person and home-based interventions). They evaluated the effects on cognitive performance and cardiorespiratory fitness, as well as the association between both. Overall, participants demonstrated a high engagement to being active, with the home-based participants demonstrating the greatest engagement to exercise. The authors found improvements in cardiorespiratory fitness. Regarding cognitive performance, they found significant improvements in verbal fluency and verbal memory. The authors suggest that engaging in a regular exercise regimen, even for a short period of two months, may promote improvements in both cardiorespiratory fitness and cognitive performance. The results suggest that the degree of exercise engagement may play a role in achieving the potential benefits of exercise (Hinchman et al.).

## Factors and environments involved in promoting exercise

It is widely accepted that exercise improves the health of people with neurological disabilities; however, this population has few opportunities to experience these benefits due to different factors and environments that present obstacles to their engagement and participation. There are limited adapted programs, accessible facilities and parks, and trained staff. Many studies have been conducted on the barriers to exercise experienced by this population for on-site programs, such as in a community park or fitness center. Recent research on exercise and disability involves the use of telehealth programs. In addition, lack of engagement in exercise has been attributed to a number of patient/family intrinsic factors and social determinants of health (SDH), which are the primary drivers of reduced access to physical activity in general.

Hansen and Allendorfer described how SDH should be taken into account in explaining lower levels of physical activity participation among people with epilepsy with cognitive impairment. Many people with epilepsy are not as active or physically fit compared to the general population. This lack of engagement in physical activity has been attributed to a number of factors such as patient or family fear of triggering a seizure, overprotection by caregivers, physical limitations, or lack of knowledge about the benefits of exercise. However, SDH, which are some of the major drivers of reduced access to physical activity in general, are rarely studied in epilepsy. The authors recommend focusing on factors related to neighborhood conditions, socioeconomic position, and accessibility to opportunities for physical activity to promote exercise for people with epilepsy and their families. Consideration of SDH allows for a greater understanding of how cognition may be both a determinant of physical activity and an outcome of environments that are conducive to physical activity in people with disabilities (Hansen and Allendorfer).

Wilroy and colleagues qualitatively explored the perceptions of people with multiple sclerosis about the potential benefits of a home exercise program, with a specific focus on benefits related to physical health and function, participation, and psychosocial health. The authors provided preliminary results regarding mobile health technologies and telehealth programs and reported on future programs to optimize critical benefits for this population. The analysis resulted in 4 themes, including: (1) improved health and function, (2) increased activity participation, (3) improved psychosocial health, and (4) optimized performance and benefits. The use of telehealth and mobile health technologies enables health professionals to deliver exercise programs that avoid barriers to exercise access (Wilroy et al.).

## Exercise and quantitative sleep spindle characteristics

Memon and colleagues determined the effects of exercise, compared with a no-exercise, sleep hygiene control group, on quantitative characteristics of sleep spindle morphology in patients with Parkinson disorder (PD). They performed a post-hoc analysis of electroencephalography derived from polysomnography collected in a previously published randomized controlled trial of the effects of exercise on sleep in PD (9). The authors showed in the current study that exercise is protective for sleep spindle density and that the change in sleep spindle density due to exercise is correlated with exercise-induced changes in memory performance. These findings have important therapeutic implications related to the utility of nonpharmacological interventions to improve sleep and cognition and suggest that exercise may influence cognitive function in PD through its effects on sleep. The current study findings indicate that high-intensity exercise, combining resistance training and body weight interval training, improves sleep efficiency and leads to improved cognitive performance in the memory domain in people with PD (Memon et al.).

The collection of papers in this Research Topic provides updates in the field of therapeutic exercise in neurological disorders as well as promising strategies and approaches for promoting increased engagement. However, there remains a continuing need to provide evidence-based research on exercise prescription and execution in neurological disorders to best guide the field moving forward.
